# Consensus Enolase of *Trypanosoma Cruzi*: Evaluation of Their Immunogenic Properties Using a Bioinformatics Approach

**DOI:** 10.3390/life12050746

**Published:** 2022-05-18

**Authors:** Alejandro Diaz-Hernandez, Maria Cristina Gonzalez-Vazquez, Minerva Arce-Fonseca, Olivia Rodríguez-Morales, Maria Lilia Cedillo-Ramirez, Alejandro Carabarin-Lima

**Affiliations:** 1 Centro de Investigaciones en Ciencias Microbiológicas, Instituto de Ciencias, Benemérita Universidad Autónoma de Puebla, 14 Sury Avenida San Claudio, Ciudad Universitaria, Puebla 72570, Mexico; alejandro.diazhernandez@viep.com.mx (A.D.-H.); lilia.cedillo@correo.buap.mx (M.L.C.-R.); 2 Herbario y Jardín Botánico Universitario, Benemérita Universidad Autónoma de Puebla, Ciudad Universitaria, Puebla 72570, Mexico; maria.gonzalezvazquez@viep.com.mx; 3 Departamento de Biología Molecular, Instituto Nacional de Cardiología “Ignacio Chávez”, Juan Badiano No. 1, Col. Sección XVI, Tlalpan, México City 14080, Mexico; minerva.arce@cardiologia.org.mx (M.A.-F.); olivia.rodriguez@cardiologia.org.mx (O.R.-M.)

**Keywords:** *Trypanosoma cruzi*, enolase, bioinformatic, chimeric peptides, molecular docking

## Abstract

There is currently no vaccine against American trypanosomiasis, caused by the parasite *Trypanosoma cruzi*. This is due to the genomic variation observed in the six DTUs of *T. cruzi*. This work aims to propose a consensus sequence of the enolase protein from different strains of *T. cruzi* and mainly evaluate its immunogenic properties at the bioinformatic level. From specialized databases, 15 sequences of the enolase gene were aligned to obtain a consensus sequence, where this sequence was modeled and then evaluated and validated through different bioinformatic programs to learn their immunogenic potential. Finally, chimeric peptides were designed with the most representative epitopes. The results showed high immunogenic potential with six epitopes for MHC-I, and seven epitopes for MHC-II, all of which were highly representative of the enolase present in strains from the American continent as well as five epitopes for B cells. Regarding the computational modeling, molecular docking with Toll-like receptors showed a high affinity and low constant of dissociation, which could lead to an innate-type immune response that helps to eliminate the parasite. In conclusion, the consensus sequence proposed for enolase is capable of providing an ideal immune response; however, the experimental evaluation of this enolase consensus and their chimeric peptides should be a high priority to develop a vaccine against Chagas disease.

## 1. Introduction

*Trypanosoma cruzi* is a flagellated protozoan parasite, discovered by Dr. Carlos Ribeiro Justiniano das Chagas in 1909 [1]; this finding is considered as a feat since it was the first time that the etiological agent and its vector were detected before the discovery of the disease [2]. The transmission of this parasite is through different routes such as blood transfusion, organ transplant, congenital via, or oral route, and it is discussed as sexual transmission; however, the most common in endemic areas is vectorial transmission, caused by hematophagous bugs [3] belonging to the Hemiptera order, Reduviidae family, and Triatominae subfamily [4]. Chagas disease is localized in 21 Latin American countries; however, in recent years, a higher incidence has been reported in the United States of America [5] as well as in some cases reported in Central Europe, Asia, and Oceania [6]; this is because of the constant migratory flows [7] and the lack of controls and screening in blood and organ donations, in which only six European countries provide the screening for Chagas disease [8]. It is estimated that 6–8 million people are infected worldwide, causing approximately 50,000 deaths per year, while 65–100 million are at risk of being infected [7], either because of their geographic location, socioeconomic status, or both. This disease is considered as a neglected tropical disease (NTD) by the World Health Organization (WHO) and some other organizations such as the Center for Disease Control (CDC), being especially common in rural areas [9].

Although *T. cruzi* has an asexual reproduction form, this parasite has a great heterogeneity with genotypic and phenotypic variation, and has therefore been classified into six highly related clades or taxonomic units, from TcI-TcVI, which have been divided based on their discrete typing units (DTU) [10,11,12]. This genetic diversity has been related to geographic distribution, pathogenesis, clinical features, and response to therapy, all well-defined by the kind of DTU [11,13].

For the treatment of Chagas disease, there are only two drugs available, Nifurtimox and Benznidazole, both developed in the last century in 1965 and 1971, respectively [14]. These treatments are not easily accessible [14,15], have high toxicity causing renal and hepatic dysfunction derived from the generation of highly reactive metabolites [16], and are indicated, especially during the acute phase, congenital, reactivations, and chronic phase in patients under ideal conditions, that is, without serious comorbidities and are commonly young (<18 years), with a probability of success around 60% [16,17].

Currently, there is no commercial vaccine capable of counteracting or preventing the disease and its dissemination in the organism. There have been considerable efforts to develop one, either with DNA platforms, attenuated vaccines, modified viruses, and bacteria, or recombinant proteins, all of them with various formulations and adjuvants [18]; however, none has been able to confer ideal protection against the disease. Some studies have suggested the combined use of vaccines and drugs to reduce treatment, and consequently, its toxicity [18]. The need to obtain important advances or alternative strategies to combat Chagas disease is evident. To this purpose, reverse vaccinology has emerged as a promising alternative [19] representing an attractive opportunity for the development of a vaccine through the analysis of proteins exposed on the surface of the parasite, thus trying to prevent infection, or at least delay its progression as well as reduce parasite congenital transmission.

Several studies have demonstrated the importance of this approach in different pathogens such as *Plasmodium falciparum* and *Ascaris suum*, showing a decrease in parasitemia by 80% and 60%, respectively, and *Candida albicans*, with IgG1 and IgG2a immunoglobulin production [20,21,22]. In a previous study, enolase from *T. cruzi* was used to show their immunogenicity, identifying a transmembrane region suggesting a surface localization; epitopes for B lymphocytes and cytotoxic T lymphocytes were also predicted, suggesting the development of humoral and cellular immune responses [23]. This protein is a multifunctional metalloenzyme with the Enzyme Commission number E.C. code 4.2.1.11, which catalyzes the reversible dehydration of D-2-phosphoglycerate to phosphoenolpyruvate [24]; enolase as an immunogen has been capable of generating a Th1 immune response (considered fundamental for intracellular parasite elimination), Th2, or a mixture of both in response to the kind of microorganism being studied (intracellular or extracellular); therefore, there is no predominance of one of these two types of the immune response, both cell populations can be expressed [21,22,25,26]. Subsequent studies detected the presence of antibodies against the recombinant enolase from *T. cruzi* (anti-rTcENO antibodies) in sera from mice experimentally immunized with rTcENO, demonstrating that the purified recombinant enolase was recognized by mouse sera. Furthermore, mice immunized with rTcENO and subsequently infected experimentally showed protection against the parasite by increasing their survival, presenting a decrease in circulating blood parasites and not presenting damage at the tissue level (cardiac and skeletal); these results indicate that rTcENO has immunogenic properties to be studied and can be proposed as a candidate for vaccine development [27].

The aim of the present work was focused on proposing a consensus sequence of the enolase protein of 429 amino acids from 15 different sequenced isolates and corresponded to different DTUs for its possible use as a vaccine. This consensus sequence was mainly evaluated for its immunogenic properties at the bioinformatic level through the analysis of epitopes, showing a high immunogenic potential with six epitopes for MHC-I and seven epitopes for MHC-II, all of them are highly representative of the American continent as well as five epitopes for B cells. Subsequently, molecular docking was performed with the membrane-associated of Toll-like receptors TLR2 and TLR4, showing a high affinity and low dissociation constant, which could lead to an innate immune response that contributes to the parasite elimination. Finally, a hypothetical chimeric construct was designed with the most representative epitopes for the Latin American population. In conclusion, the enolase consensus sequence would have the capacity to provide an ideal immune response for people at risk of infection with Chagas disease in Latin America and the world; similar properties could have chimeric proteins, which need further study and experimental evaluation to verify whether they confer protection to most of the *T. cruzi* strains of Chagas disease.

## 2. Materials and Methods

### 2.1. Generation of Enolase Clusters

Based on the coding gene for the enolase from *T. cruzi* H8, corresponding to DTU I, with access number to GenBank KC862322.1 and a length of 1151 bp [23], a sequence search was performed in general and specialized databases such as the National Center for Biotechnology Information (NCBI) and the specialized database on pathogens of the family Trypanosomatidae (TriTrypDB) [28]. Subsequently, the *T. cruzi* CL Brener DTU VI enolase gene, with 1290 bp and accession number XM_814607.1, was used as a template, since it is the best characterized DTU. In the NCBI megablast programs, sequences with the highest percentage of identity, an e-value of 0.0, and coverage greater than 85% were selected; in the TriTrypDB, sequences with an e-value of 0.0, a score greater than 2000, an identity greater than 95%, and 0 gaps in the sequences were obtained. The sequences were classified into their different DTUs and were translated from DNA to amino acids using standard genetic coding with the EMBOSS Transeq platform [29].

### 2.2. Consensus Sequence and Homology Modeling

The analysis of the 15 translated sequences was performed with the Jalview program [30] by analyzing them in Clustal Omega multiple alignments and examining the conservation, mutations, gaps, and biochemical properties of the sequence along all of the available DTUs. Subsequently, a consensus sequence was obtained, and from this, homology modeling was performed with the SWISS-MODEL platform [31] using the crystallized protein as a template, which was deposited in the Protein Data Bank (PDB) database [32] with code 4G7F, belonging to the DTU VI CL Brener.

In homology modeling, features such as the global model quality estimation (GMQE) score, qualitative model energy analysis (QMEAN) score, Cβ, and all-atom interaction potential, solvation potential, and torsion angle as well as the local quality values for each amino acid, compared to the annealing, and a comparison graph with experimentally obtained structures of similar sizes were analyzed. The modeling was also evaluated using Ramachandran plots to validate the quality of the modeling through the MolProbity 4.4 program [33]. Finally, the model was analyzed using the UCSF Chimera program [34], where both structures, the 4G7F crystallized *T. cruzi* CL Brener DTU VI enolase and the modeling one, were superimposed to identify their differences at the sequence and structural level.

### 2.3. Analysis of the Physicochemical and Immunogenic Properties and Prediction of Epitopes Associated with B and T cells

The ProtParam bioinformatics service [35] for the physicochemical characteristics of the sequence was used; the web-based vaccine target design program Vaxign 2.0 [36] in combination with the Immune Epitope Database (IEDB) [37] was employed to evaluate the immunogenic properties as well as the reference human leukocyte antigens (HLA) that could recognize the consensus sequence, and for the prediction of Major Histocompatibility Complex I and II (MHC-I and II) epitopes mainly associated with the Latin American population, selecting only epitopes with a *p*-value ≤ 0.01 for both cases. The selection and validation of epitopes were first performed by their representativeness in the HLA’s supertypes, and then by the presence of proteasomal cleavage sites for the case of MHC-I through the NetChop 3.1 platform [38].

For the prediction of the B cell epitope prediction, their selection and validation were performed with the programs BepiPred 2.0 [39] and Discotope 2.0 [40] using linear and structural analysis, respectively, to identify the protein regions most likely to be recognized by antibodies. To better identify the MHC-I and -II epitopes as well as those corresponding to B cells, visualizations were performed by the UCSF Chimera program, highlighting their respective regions.

### 2.4. Molecular Docking and Interaction Analysis

Molecular docking studies were performed to analyze the binding affinity between the modeling protein and membrane-associated TLR2 (PDB ID: 2Z7X) [41] and TLR4 (PDB ID: 4G8A) [42]. Docking was performed with the HDOCK server [43], which reported more than 150 models ranked by docking score, and the one with the best score was selected for both cases. Once the models were selected, the file was downloaded in PDB format and uploaded to the PRODIGY platform [44], which indicates the Gibbs free energy (ΔG) as well as the dissociation constant (Kd). Finally, the analysis of the interactions and visualization of the protein–receptor complex was performed using PDBsum [45].

### 2.5. Protein Chimera Construction

Two protein chimeras containing the predicted epitopes were manually designed, mainly for T cells, six for MHC-I, and seven for MHC-II. The connector peptides to bind these epitopes were used: GPGPG and GGGS for their high flexibility and EAAAK for their rigidity, respectively. Both chimeras were made up of a type 0 Cap (Cap) (m7G(5′)pppN1pN2p), an untranslated region (UTR) sequence with the coding gene for β-globin in the 5′ region; an EAAAK linker coding sequence; a Kozak sequence immediately followed by the cytotoxic T lymphocyte (CTL) and helper T lymphocyte (HTL) epitope coding sequence separated by the GGGS and GPGPG linkers, respectively; an EAAAK linker coding sequence attached to a UTR with the gene for α-globin in the 3′ region; and finally a poly-A tail of 120–150 bases.

### 2.6. Characterization of Protein Chimeric Constructs

The physicochemical features were evaluated in the ProtParam [35] server, and the prediction of the secondary structure was performed with the PsiPred tool [46] and compared with the Phyre v2.0 server [47].

The protein chimeric constructs were evaluated to determine their immunogenicity using VaxiJen v4.0 [48] (http://www.ddg-pharmfac.net/vaxijen/VaxiJen/VaxiJen.html, accessed on 10 April 2022) and was used at 0.5 thresholds to ensure the properties of the target epitopes. Additionally, the prediction of the potential allergenicity was calculated by AllerTOP v2.0 [49]. Finally, the ToxinPred tool [50] was used to verify the linear peptides’ potential toxicity.

The tertiary structures for chimeric constructs were predicted by AlphaFold2 [51]; this program can generate accurate 3D models of proteins from the primary sequence without utilizing templates [52]. Moreover, AlphaFold introduced an improved confidence measure, the predicted local Cα distance difference test (pLDDT). Molecular docking was performed with the GRAMM-X server v1.2.0 [53]. Once the models were selected, the files were downloaded in the PDB format and uploaded to the PRODIGY platform. Finally, the analysis of the interactions and visualization of the protein–receptor complex was performed using PDBsum.

## 3. Results

### 3.1. T. Cruzi Enolase Clusters and Consensus Sequence

A total of 15 coding sequences for the *T. cruzi* enolase were found, 13 from TriTrypDB and two from the NCBI (Table 1). The 15 sequence-links and their references belonged to different DTUs, except for DTU IV in which no sequences were found, are shown in Table S1.

**Figure 1:**
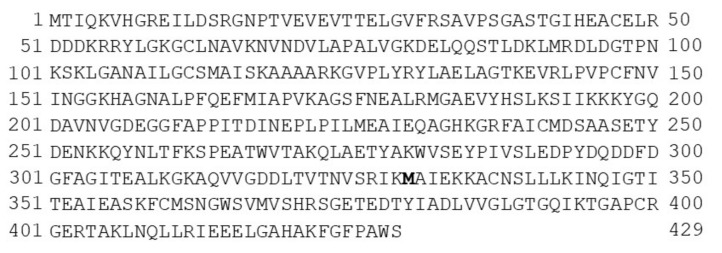
Consensus sequence of the *T. cruzi* enolase with a length of 429 amino acids. The constant mutation (T330M) at the 330 position of the amino acid sequence was present in eight of the 15 enolase sequences analyzed with respect to the reference enolase CL Brener strain (this mutation is represented in bold).

Once identified, the 15 coding sequences for the *T. cruzi* enolase were translated into amino acids using the EMBOSS Transeq, based on the universal genetic code, obtaining a total of 429 amino acids for all of the sequences, except for H8 (DTU I), which coded for 384 amino acids due to the smaller number of nucleotides (1151 bp). Most of the sequences showed high identity and conservation throughout the amino acid sequence. The Marinkellei B7 strain belonging to DTU VI was the sequence that showed more punctual mutations in 14 positions, followed by strains, which only presented a single mutation in the 308 position. It was also possible to observe a constant mutation at the 330 position, in which seven DTUs had threonine (T) and eight had methionine (M). Due to this last variation, the resulting consensus sequence showed M (in bold) at the 330 position out of a total of 429 amino acids (Figure 1).

### 3.2. Modeling by Homology of Enolase Consensus Sequence

Based on the consensus sequence of 429 amino acids, homology modeling was performed using the crystallized enolase of *T. cruzi* CL Brener DTU VI PDB: 4G7F as a template, with an identity of 99.77% between both proteins. The registered values that estimate the quality of the modeling were the QMEAN (degree of nativity or authenticity) of −0.28, good Cβ values, the interaction between all the atoms of the structure, the solvation potential and torsion angle, and a GMQE of 0.96 and the local quality values were also obtained for each amino acid. Likewise, a comparison plot is shown, with a model protein score of Z-score <1, which was compared with the structure scores experimentally obtained of similar sizes using the normalized QMEAN value (Figure S1) as a reference.

**Figure 2:**
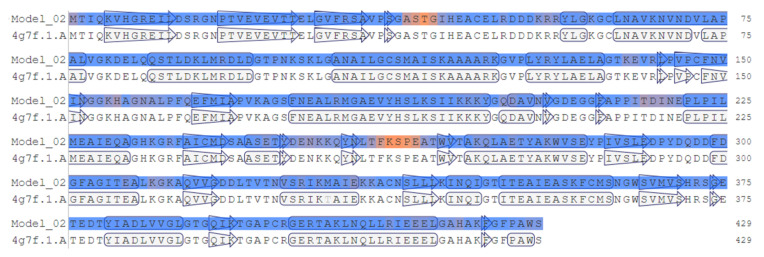
Linear alignment between the enolase consensus sequence (Model_02) and 4G7F crystallized enolase sequence using the QMEAN color scheme (orange for low and blue for ideal scoring regions).

**Figure 3:**
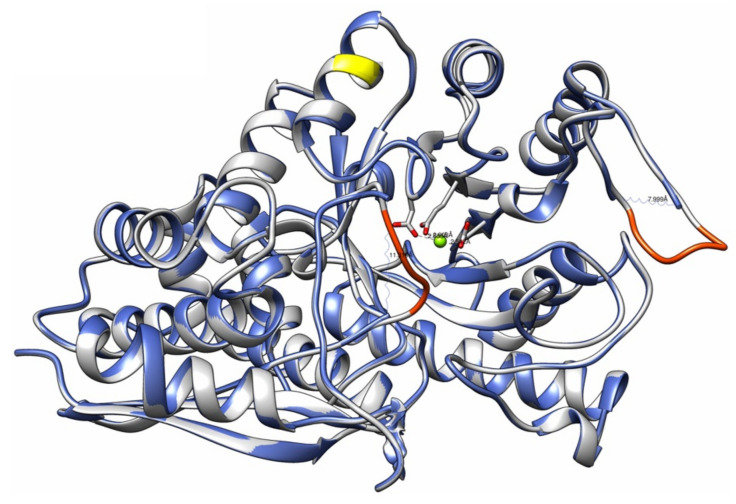
Superposition of both protein structures: modeling enolase consensus sequence (grey) and 4G7F crystallized enolase (blue). The yellow and orange regions show the methionine substitution at the 330 position and the missing regions in the 4G7F structure, respectively. Visualization of the structures was performed with the UCSF Chimera program [34].

The modeling enolase consensus sequence (Model_02) and the original structure corresponding to the 4G7F crystallized *T. cruzi* CL Brener DTU VI enolase were analyzed with the modeling structure using the UCSF Chimera program. A root-mean-square deviation (RMSD) value of 0.068 by alignment (Figure 2) and superimposition between both sequences were obtained. The structural alignment again showed an identity of 99.77%; however, it also showed a region of 10 missing amino acids (orange color) within the crystallized structure, corresponding to regions 39–42 and 260–265, with amino acids “A, S, T, G and T, F, K, S, P, E, respectively, as well as the only amino acid change between both sequences at the 330 position from methionine to threonine (Figure 3) (yellow color). The modeling structure was also analyzed using four Ramachandran plots (General, Glycine, Proline, and Pre-proline) (Figure S2), obtaining MolProbity values, overall Ramachandran score, and deficient junctions in the structure (Table 2).

### 3.3. Analysis of Physicochemical and Immunogenic Properties

The physicochemical properties were analyzed by ProtParam; this analysis showed crucial values such as molecular weight, half-life hours in different organisms, and stability (Table 3). The immunogenic characteristics were analyzed by Vaxign 2.0, in which the adhesion, location, and Vaxign-ML score values were obtained (Table 3).

**Figure 4:**
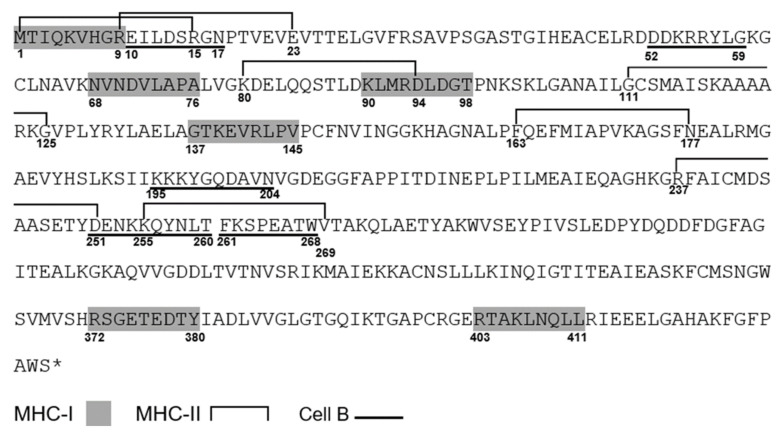
The modeling enolase consensus sequence of 429 amino acids with the highlighted predicted epitopes. MHC-I epitopes are shown in the grey shadow, MHC-II epitopes are shown with superior brackets, some of them overlapping, and B cell epitopes are underlined. The symbol * indicates the termination of the open reading frame.

The Vaxign 2.0 and IEDB were used to predict the MHC-I/II epitopes using reference HLAs mainly for the Latin American population. More than 1500 possible combinations were obtained for each MHC, and based on their *p*-value ≤ 0.01, six epitopes were selected for MHC-I and seven for MHC-II, showing three regions overlapping with epitopes for MHC-I (Figure 4). The predicted epitopes corresponding to MHC-I were analyzed through the NetChop 3.1 platform, in which the presence of proteasomal cleavage in these regions was identified (Table 4). Regarding the epitopes for B cells, the BepiPred 2.0 and Discotope 2.0 platforms were used separately to perform linear and structural analysis, respectively, where both showed five similar regions with a high probability of being recognized by antibodies (Table 5), with three regions overlapping with one or more epitopes for HLAs.

**Figure 5:**
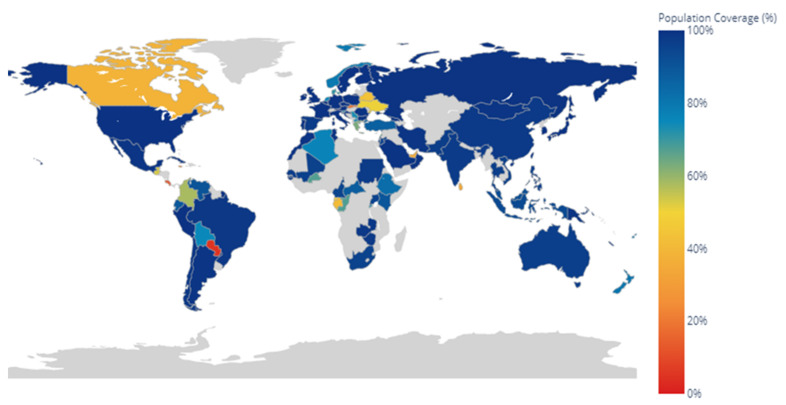
The hypothetical global protection map using the complete modeling enolase consensus sequence protein as a vaccine.

The Vaxign 2.0 analysis included a world map with the percentage of predicted protection for each country based on the reference HLAs, the epitopes of the modeling protein that can bind to the MHC-I or II of T cells, and the information available in the IEDB database, showing most of the regions with 100% predicted protection (Figure 5).

### 3.4. Molecular Docking and Protein–Receptor Interactions

Molecular docking analysis between the modeling enolase and TLR2 and/or TLR4 reported more than 150 possible models per receptor based on the arbitrary docking score system. The best scoring ones were selected, being −249.7 and −262.99, respectively; both models were downloaded and sent to the PRODIGY server to indicate the Gibbs free energy (ΔG) and dissociation constant (Kd) at 25 °C, both showing a high probability of binding between the receptor and the modeling enolase protein (Table 6).

**Figure 6:**
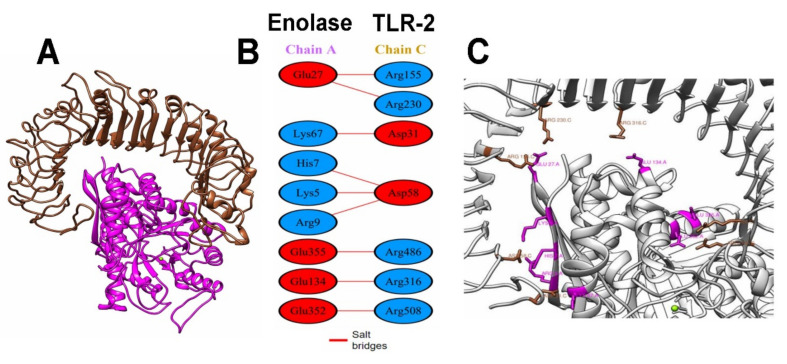
(**A**) Interaction between the TLR2 (brown) and enolase consensus (magenta); (**B**) PDBsum analysis, schemes with the type and number of interactions between the residues of the A chain (modeling enolase) with the C (TLR 2) with the same formatting; (**C**) contact points for salt-bridges between the TLR2 (brown) and enolase consensus (magenta).

For better visualization of the interactions between the modeling enolase protein and TLRs, the docking files generated in HDOCK were analyzed using the PDBsum platform. The analysis of the modeling enolase protein-coupled to TLR2 showed nine salt-bridge interactions between the A chain (consensus enolase) and the C chain (TLR2) as well as eight and seven interacting residues, respectively, in an area of 2003 and 1884 Å^2^ for the A and C chains (Figure 6).

**Figure 7:**
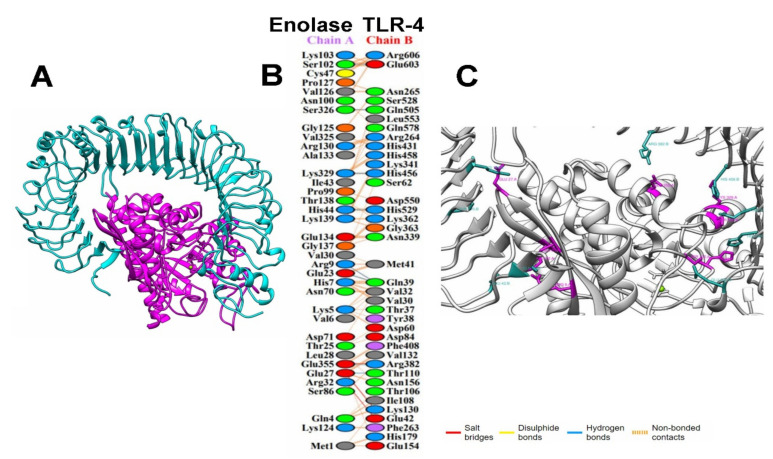
(**A**) Interaction between the TLR4 (cyan) and enolase consensus (magenta); (**B**) PDBsum analysis, schemes with the type and number of interactions between residues of the A chain (modelling enolase) with the C (TLR4) with the same formatting; (**C**) contact points for salt-bridges between TLR4 (cyan) and enolase consensus (magenta).

For the interaction of enolase with the TLR4 receptor, the analysis showed that this interaction was confirmed by the presence of three salt-bridges, eight hydrogen bridges and 257 weak bonds, as well as 36 residues of the A chain (enolase consensus) interacting with 38 residues of the B chain (TLR4, in an area of 1980 and 1950 Å^2^ for the A and B chains, Figure 7).

### 3.5. Enolase Chimera Epitope-Based

**Figure 8:**
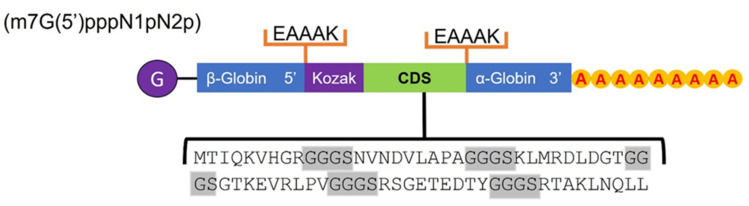
The general structure of the protein chimera coding constructs with the predicted epitope coding sequence for MHC-I; the coding sequences corresponding to the connectors are shown in the gray shadow.

A manual design of two chimeras with the information corresponding to the epitopes for MHC-I/II was carried out. The structure of the first chimera in the 5′ to 3′ direction presented a 0-type cap, a UTR sequence with the necessary information for β-globin, an EAAAK coding sequence rigid protein connector, a Kozak sequence immediately followed by six CTL epitopes for MHC-I detected with the Vaxign 2.0 and separated by GGGS coding sequence connectors, and again, an EAAAK coding sequence connector attached to a UTR with the gene corresponding to the α-globin in the 3′ region, attached to a 120–150 bp poly-A tail (Figure 8).

**Figure 9:**
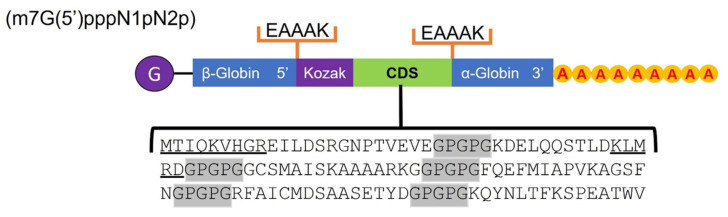
The general structure of the protein chimera coding constructs with the predicted epitope coding sequence for MHC-II; regions belonging to MHC-I are underlined, and the coding sequences corresponding to connectors are shown in the grey shadow.

The second chimera was similar in design, except for the seven HTL epitopes for MHC-II that were also detected through the bioinformatics analysis; these were separated by GPGPG connectors between each other (Figure 9).

### 3.6. Physicochemical Features, Secondary Structural Analysis, and Modelling of Protein Chimeric Constructs

To determine the physicochemical features of the protein chimeric constructs, the ProtParam server was used to analyze both constructs; the main results are shown in Table 7. Moreover, the results indicate that both chimeric constructs are stable to show an instability index for the MCH-I construct of 36.10 and for the MHC-II construct of 34.65. The negative GRAVY indexes of −0.582 and −0.508 for the MHC-I and MHC-II chimeric constructs are indicative of hydrophilic and soluble proteins.

The secondary structure of the chimeric constructs for MHC-I indicated a 74 amino acid long construct involving 16% strands, 27% helices, and 59% random coils. The results for MHC-II indicated a 123 amino acid long construct involving 46% strands, 12% helices, and 32% random coils (data no shown). To obtain a 3D protein chimeric construct (Figures S4 and S5), we used the AlphaFold server, which has a high capacity to solve a sequence without requiring a specific template. The results obtained for both MHC-I/MHC-II constructs were compared with those obtained by the Phryre2 server. This server considerably used the *Trypanosoma brucei* enolase template, which showed confidence values and identity percentages for the MHC-I construct of 92.1% and 83%, respectively, and for the MHC-II construct of 99.6% and 89%, respectively, indicating that the enolase specificity was maintained in these designed protein chimeras.

### 3.7. Chimeric Constructs: Antigenicity, Allergenicity Profiling, and Proteasomal Cleavage

The antigenicity of both chimeric constructs was high, with 0.7570 and 0.5691 scores for the MHC-I and MHC-II constructs, respectively, as predicted by VaxiJen and with a threshold of 0.5, suggesting that the chimeric constructs are immunogenic and can trigger a strong immune response. The AllerTOP tool classified both chimeric constructs as non-allergenic in humans and predicted that the constructs were non-toxic. On the other hand, the identification of possible proteasomal processing sites was performed using the NetChop server, in which 12 sites in the MHC-I protein chimera construct were identified with a threshold of 0.9.

### 3.8. Molecular Docking of Protein Chimeric Constructs and Protein-Receptor Interactions

Molecular docking analysis between the MHC-I/MHC-II protein chimeric constructs and TLR2 or TLR4 were performed in the GRAMM-X server. The best models were selected, downloaded, and sent to the PRODIGY server to predict the Gibbs free energy (ΔG) and dissociation constant (Kd) at 25 °C; the results showed a high probability of binding affinity between the TLR and the protein chimeric constructs (Table 8).

**Figure 10:**
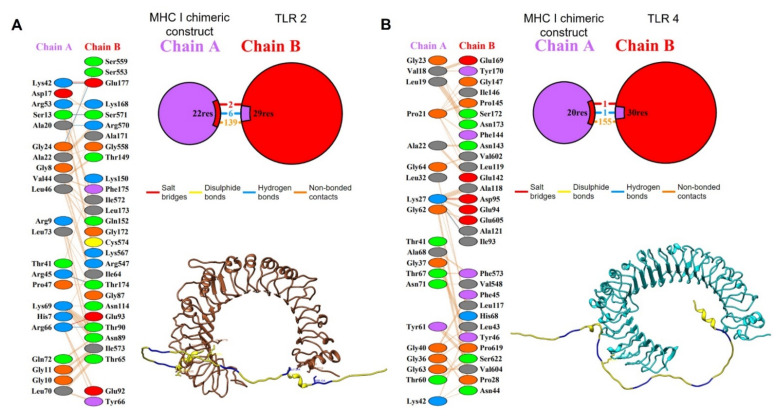
Interactions of TLR2 (brown) and TLR4 (cyan) with the modeled chimeric structure (yellow for epitopes and blue for linkers, respectively). (**A**) The PDBsum analysis, schemes with the type and number of interactions between the residues of the A chain (modeled chimeric structure) with the B chain (TLR2) and contact points for salt-bridges, hydrogen bonds, and non-bonded contacts, between the residues of the A chain (modeled chimeric structure) and the B (TLR2) are shown; (**B**) The PDBsum analysis, schemes with the type and number of interactions between the residues of the A chain (modelled chimeric structure) with the B chain (TLR4) and the contact points for the salt-bridges, hydrogen-bonds, and non-bonded contacts between the residues of the A chain (modelled chimeric structure) and the B (TLR4) are shown.

For better visualization of the interactions between the MHC-I/MHC-II protein chimeric constructs and TLRs, the molecular docking files generated in GRAMM-X were analyzed using the PDBsum platform. The analysis of the MHC-I protein chimeric construct coupled to TLR2 showed the presence of two salt-bridges, six hydrogen-bridges, and 139 weak bonds as well as 22 residues of the A chain (MHC-I construct) interacting with 29 residues of the B chain (TLR2, in an area of 1385 and 1282 Å^2^ for the A and B chain (Figure 10A).

For the interaction of the MHC-I protein chimeric construct with the TLR4 receptor, the analysis showed the presence of one salt-bridge, one hydrogen-bridge, and 155 weak bonds as well as 20 residues of the A chain (MHC-I construct) interacting with 30 residues of the B chain (TLR4, in an area of 1267 and 1034 Å^2^ for the A and B chain (Figure 10B).

**Figure 11:**
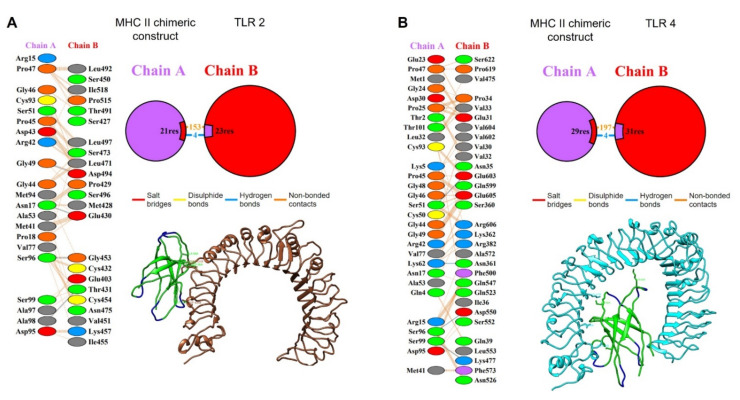
Interaction of TLR2 (brown) and TLR4 (cyan) with the modeled chimeric structure (green for epitopes and blue for linkers, respectively). (**A**) The PDBsum analysis, schemes with the type and number of interactions between the residues of the A chain (modeled chimeric structure) and the B (TLR2) with contact points for hydrogen bonds and non-bonded contacts, between the residues of the A chain (modelled chimeric structure) and the B (TLR2) are shown; (**B**) The PDBsum analysis, schemes with the type and number of interactions between residues of the A chain (modelled chimeric structure) with the B (TLR4) with contact points for hydrogen bonds and non-bonded contacts, between the residues of the A chain (modeled chimeric structure) with the B (TLR4) are shown.

The interaction between the MHC-II chimeric construct and TLR2 showed the presence of four hydrogen bridges and 153 weak bonds as well as 21 residues of the A chain (MHC-II construct) interacting with 23 residues of the B chain (TLR2, in an area of 1161 and 1143 Å^2^ for the A and B chains, respectively (Figure 11A).

For the interaction of the MHC-II protein chimeric construct with the TLR4 receptor, the analysis showed that this interaction was confirmed by the presence of four hydrogen bridges and 197 weak bonds as well as 29 residues of the A chain (MHC-II construct) interacting with 31 residues of the B chain (TLR4, in an area of 1555 and 1492 Å^2^ for the A and B chains (Figure 11B).

## 4. Discussion

Several studies have validated the use of bioinformatics tools in the search for new vaccines from the use of TriTrypDB for the reliable search of new antigens in *T. cruzi* [54]. Taking into account the above and based on the TriTrypDB and NCBI databases, 13 and two sequences of the enolase were obtained, respectively, belonging to almost all known DTUs except for DTU IV, which can be explained by the constant association of this variant with dogs as well as other domestic mammals in highly isolated regions such as the Paraguayan Chaco [55], and therefore, the lack of study at the genome sequencing level. Later, the sequences were translated into amino acids, resulting in 429 amino acids for 14 sequences of 1290 bp, while the initial reference sequence corresponding to the H8 variant has 1151 bp, which codes for 384 amino acids; therefore, seeking greater reliability, the reference strain became the one corresponding to CL Brener, since it is the most studied variant worldwide [56].

To obtain an enolase consensus sequence, the multiple alignments based on Clustal Omega showed that the Marinkellei B7 variant strain of *T. cruzi* showed more point mutations, with 14 throughout the alignment (data not shown). It is also important to mention that a constant mutation was identified at the 330 position, in which seven DTUs had T and eight had M while the consensus sequence had an M of a total of 429 amino acids. The homology modeling used as a template for the crystallized structure of the *T. cruzi* CL Brener enolase (PDB: 4G7F) (DTU VI) showed an identity of 99.77% at the sequence level, and QMEAN value of −0.28 and GMQE of 0.96, which indicates the degree of nativity or authenticity and quality of the global model, respectively. In this regard, the homology modeling performed by Vedamurthy G. et al., (2019) [50] on the same platform showed the following values GMQE: 0.72, identity: 46.89%, and overall Ramachandran of 98.5%, unlike the enolase consensus obtained here, whose values were GMQE: 0.96, identity of 99.77%, and overall Ramachandran of 96.49%. These data indicate that the modelling is highly reliable, and data are confirmed by a comparison plot shown in Supplementary Figure S1, which displayed the experimentally obtained protein structure data. There were two regions less than 0.6 (poor quality), corresponding to regions 39–42 and 260–265 with amino acids “A, S, T, G and T, F, K, S, P, E”, respectively, visualized under the 3D representation and the QMEAN coloring scheme in Figure 3. Their absence did not seem to affect the interaction with TLRs, since no interactions have been reported for these regions, and they do not cover any of the detected epitopes. Even with this absence at the structural level, the superposition of both structures showed an RMSD of 0.068, which also validated the similarity between both models, and only one change was shown at the amino acid level corresponding to the 330 position, which did not seem to affect the alpha-helix structure and is shown in the yellow color in Figure 3. Finally, the analysis of the structure with the Ramachandran graphs showed an overall value of 96.49% compared to an ideal of 98%; however, this value can be explained by the absence of the mentioned regions, which would be missing the amino acids of the model, among them, a glycine that due to its flexibility could contribute to a theoretical conformation, which results in a steric hindrance, and according to the MolProbity score, an overall value of 0.93 is indicated, and that the closer to zero, but lower than the template resolution (2.40 Å), would be a better value.

The analysis of the physicochemical properties indicated an expected molecular weight as well as hours of half-life in different organisms and a stability of 39.77, both being ideal for purification processes [35,57]. Regarding the immunogenic characteristics, an expected cytoplasmic location and a Vaxign-ML score of 91.7 were obtained, indicating the probability of the enolase consensus to be a good vaccine antigen based on the data banks of the experimentally tested immunogens [36]. The predicted epitopes for MHC-I and -II resulted in more than 1500 initial combinations for both cases using the reference HLAs for the Latin American population: initially choosing those with a *p*-value ≤ 0.01, then discarding them for their representativeness in HLA supertypes, and finally, for the presence of proteasomal cleavage sites in the case of MHC-I. The results were six epitopes for MHC-I and seven epitopes for MHC-II, which are shown in linear form in Figure 4. These results indicate that three MHC-II epitopes overlapped with regions of the MHC-I epitopes, which could help to generate a complete humoral and cellular immune response. Epitopes for B cells were also identified by linear and structural analysis, where both showed five regions with a high probability of being recognized by antibodies, and also showed splicing in three regions with one or more epitopes corresponding to HLAs. To have a better perspective and identification of the epitopes for MHC-I and -II as well as those corresponding to B cells, 3D visualizations were performed through the UCSF Chimera program, highlighting the respective regions shown in Figure S3, where it can be appreciated that most of them are exposed in the structure of the enolase consensus, which would facilitate their recognition. The analysis by Vaxign 2.0 also showed a world map with the percentage of predicted protection for each country or region based on the reference HLAs used, the epitopes of the modeling protein that can bind to the MHC-I or -II of T cells, and the information available in the IEDB database. Regarding this, good protection was observed, mainly for a large part of Latin America, with most of its regions showing 100% predicted coverage. The analysis of the immunogenic properties and epitope prediction performed by Ong E. et al., (2020) [58] showed a review of several SARS-CoV-2 vaccines in development through the Vaxign platform, demonstrating Vaxign-ML values >90 for three proteins, while another study carried out by Khan M. et al., (2021) [59] showed the obtention of 18 epitopes through the IEDB platform focused on specific populations according to their HLA. These platforms are par excellence the most widely used in the design and research of new antigens; however, the recent development of the Vaxign 2.0 platform combines the two previous platforms showing values validated by both servers.

Once the regions of interest were defined, we proceeded to perform a molecular docking analysis between the modeling protein and TLR2 and TLR4, choosing the best docking based on the docking score (Figure 6 and Figure 7). These assays yielded values of ΔG: −18.1 and Kd: 5.0 × 10^−14^ for TLR2 as well as ΔG: −18.6 and Kd: 2.5 × 10^−14^ for TLR4. An analysis of molecular docking performed by Saha R., et al., (2021) [60] reported values for ΔG of up to −19.9, similar to those obtained in this work; these data suggest a high probability of binding between the receptor and the modeling enolase protein; therefore, a probable activation of the innate immune response at the level of membrane-associated receptors could be happening, which could contribute to the elimination of the parasite in the host through the production of proinflammatory cytokines such as TNF-α and IFN-γ, which in turn can stimulate the production of reactive nitrogen species [61,62].

Finally, the analysis performed with PDBsum showed more detailed information and diagrams of the interactions between both proteins, showing only nine salt-bridge interactions between chain A (enolase consensus) and chain C (TLR2). In the second analysis, three salt-bridges, eight hydrogen-bridges, and 257 weak bonds between chain A and chain B (TLR4) were shown. This detailed analysis of the bonds in the docking quantitatively and qualitatively describes the binding, being especially high for TLR4, with numerous electrostatic and van der Waals type interactions. This information reinforces the probable interaction necessary to activate the receptors since these interactions are fundamental for the stabilization of the protein complex, which leads to an innate immune response that can activate an immediate adaptive immune response [63,64].

Of the two protein chimeras that could be proposed as potential mRNA vaccines, the first one, with epitopes for MHC-I, can be seen in Figure 8, consisting mainly of a type 0 cap that helps to increase immunogenicity and contains a Kozak sequence to facilitate recognition and thus translation initiation. Then, the coding DNA sequence or CDS region contains the epitopes separated by a GGGS connector indicated by its flexibility in epitopes of up to nine amino acids. There are also two UTR regions flanking the chimera with the coding genes for β-globin and α-globin, which helps to generate adequate stability and increase their translation [65,66,67]. In the case of the second chimera, the same structure was proposed, except for the epitopes; in this case, they corresponded to MHC-II, but due to their length, some of them overlapped with epitopes for MHC-I, as shown in Figure 9, separated by GPGPG connectors that provide greater flexibility, which is necessary in longer epitopes. The design of both chimeras was intended to be in a general way, since further in silico evaluations are needed to propose a more detailed structure; however, the epitopes proposed here show considerable potential for further study and proposal as a vaccine. The design of both protein chimeras containing the most representative epitopes of the enolase consensus was performed manually, since it is a proposal based on structures designed and validated by different authors such as Saadi M. et al., (2017) [68]; Srivastava S. et al., (2018) [69]; Michel-Todó L. et al., (2019) [54]; He J. et al., (2020) [70]; Khan M. et al., (2021) [59]; and Saha R. et al., (2021) [60] following recommendations focused on the use of fusion peptides, stabilizing genes, and translation enhancers as well as the use of the zero-type cap to enhance the immune response. The proposed MHC-I/MHC-II proteins’ chimeric constructs proved to be antigenic and not allergenic or toxic, so its use in assays as immunogens is possible. On the other hand, the modeling of these protein chimeric constructs was carried out with the AlphaFold server, which has proven to be the best method for modeling compared to others and generates results very similar to those obtained by crystallography [51].

Although the analyses with bioinformatics tools by these different research groups are not exclusive to *T. cruzi*, they cover other models and validate their use; this is an important approach in the development of vaccines and the search for new antigens, which need to be complemented through research and validation in vitro and in vivo.

The ideal type of immune response for *T. cruzi* elimination in humans is not yet fully elucidated, but several studies have suggested a Th1 immune response as fundamental for parasite elimination [71] as well as a humoral immune response that contributes to the production of proinflammatory cytokines such as TNF-α, IL-2, and IFN-γ [72], which in turn stimulate the production of reactive oxygen species [71] as well as the production of antibodies for the elimination of the parasite in the bloodstream [72]. The results obtained in this work identified the selected epitopes and in particular, the modeling consensus of enolase, as serious candidates to provide protection against Chagas disease through the activation of a Th1/Th2 immune response, presenting so far, the ideal theoretical immunogenicity against almost all known strains of *T. cruzi*. However, further complementary in silico, in vitro, and in vivo studies are required to confirm that the proposed enolase consensus or chimeric peptides can confer immunity against *T. cruzi* infection.

## 5. Conclusions

The high genetic diversity in the different DTUs of *Trypanosoma cruzi* has been an obstacle in obtaining an immunogen capable of conferring protection against the different DTUs. In this work, the possibility of obtaining an enolase consensus sequence, which is a highly conserved protein, and that through a bioinformatic approach we could obtain a consensus sequence that could generate an adequate immune response against all DTU, were analyzed. This enolase consensus would be capable of generating effective immune responses against the different DTUs in this parasite. Moreover, protein chimeric constructs were generated and evaluated, showing that they could become very good strategies in developing a vaccine against Chagas disease, so their experimental analysis as well as that of the enolase consensus should be a priority to be evaluated.

## Figures and Tables

**Table 1:** Fifteen *T. cruzi* strains with identical sequences to enolase from H8 and CL Brener *T. cruzi* were found in TriTrypDB and NCBI.

DTU	*T. Cruzi* Strains
I	Dm 28c, Jrcl 4, Sylvio X10/1, Brazil A4, H8 ^1^
II	Esmeraldo, Y, Yc6
III	231
IV	
V	Bug2148
VI	TCC, Marinkellei B7, TulaCl2, CL Brener Non-Esmeraldo-like, CL Brener ^1^

^1^ Sequence from NCBI.

**Table 2:** Values by MolProbity of the modeling protein.

Scores	Obtained Values	Ideal Values
MolProbity	0.93	0
Clash	0.61	0
Ramachandran favored	96.49%	>98%
Ramachandran outliers	0.70%	<0.2% *
Rotamer outliers	0.87%	<1% *
Bad bonds	1/3317	0
Bad angles	20/4483	0

* Values are reported as errors due to a resolution of less than 3 Å in the template protein, as indicated by the program.

**Table 3:** Values by ProtParam and Vaxign 2.0 for the modeling protein.

Program	Obtained Values	Ideal Values
ProtParam	46,474.05 Da (Molecular weight)	N/A
	5.92 (Theoretical isoelectric point)	N/A
	50 (Positively charged residues Arg + Lys)	N/A
	55 (Negatively charged residues Asp + Glu)	N/A
	C_2049_H_3279_N_563_O_631_S_18_ (Formula)	N/A
	6540 (Total atoms)	N/A
	30, >20, and >10 (Half-life hours in mammals, and in vitro, yeast and bacteria, respectively)	N/A
	39.77 (Instability index)	<40
Vaxign 2.0	0.2 (Adhesion)	1
Vaxign 2.0	1.0 (Cytoplasmic location)	1
Vaxign 2.0	91.7 (Vaxign–ML)	≥90

N/A = Not applicable.

**Table 4:** Enolase predicted epitopes by Vaxign 2.0 for MHC-I and -II.

MHC-I Sequences	Proteasomal Cleavage	HLA Supertypes	MHC-II Sequences	HLA Supertypes
MTIQKVHGR	4	A68, A33, A31, A11	GCSMAISKAAAARKG	DPA1, DPB1, DRB5, DRB1, DRB3
GTKEVRLPV	4	A68, A30, A01	KDELQQSTLDKLMRD	DPA1, DPB1, DRB5
GTKEVRLPV	3	A02, A68	KQYNLTFKSPEATWV	DRB5, DRB1
KLMRDLDGT	4	A02	RFAICMDSAASETYD	DRB1
RSGETEDTY	2	A30, B58, B15	REILDSRGNPTVEVE	DRB1, DQA1, DQB1
RTAKLNQLL	4	A24, B58, B57	MTIQKVHGREILDSR FQEFMIAPVKAGSFN	DRB3, DQA1, DQB1, DPA1, DPB1 DRB1, DQA1, DQB1

**Table 5:** Predicted B cell enolase epitopes by BepiPred 2.0 and Discotope 2.0.

Sequence	Length	Overlapping with HLA Epitopes
EILDSRGN	8	Yes (2)
DDKRRYLG	8	No
KKKYGQDAVN	10	No
DENKKQYNLT	10	Yes (2)
FKSPEATW	8	Yes (1)

**Table 6:** Values by HDOCK and PRODIGY to predict the probability of the binding affinity between the modeling enolase protein and membrane-associated TLR2 and TLR4.

Receptor (PDB)	Docking Score	Affinity Energy ΔG (kcal/mol)	Dissociation Constant K_d_ (M) 25 °C
TLR 2 (2Z7X)	−249.7	−18.1	5.0 × 10^−14^
TLR 4 (4G8A)	−262.99	−18.6	2.5 × 10^−14^

PDB = Protein Data Bank.

**Table 7:** Physicochemical properties of the MHC-I/MHC-II protein chimera constructs.

Physicochemical Properties	MHC-I Chimeric Construct	MHC-II Chimeric Construct	Ideal Values
Molecular weight	7342.12 Da	12,765.42 Da	N/A
Theoretical isoelectric point	9.39	6.56	N/A
Positively charged residues Arg + Lys	7	13	N/A
Negatively charged residues Asp + Glu	9	13	N/A
Formula	C_304_H_510_N_100_O_107_S_2_	C_556_H_873_N_157_O_174_S_7_	N/A
Total atoms	1023	1767	N/A
Half-life hours in mammals and in vitro, yeast and bacteria, respectively	30, >20 and >10	30, >20 and >10	N/A
Instability index	36.10	34.65	<40

N/A = Not applicable.

**Table 8:** Values by PRODIGY to predict the probability of the binding affinity between the MHC-I/MHC-II protein chimeric constructs and the membrane-associated TLR2 and TLR4.

Protein Chimeric	Receptor (PDB)	Affinity Energy ΔG (kcal/mol)	Dissociation Constant K_d_ (M) 25 °C
MHC-I	TLR 2 (2Z7X)	−14.3	3.1 × 10^−11^
	TLR 4 (4G8A)	−13.9	6.0 × 10^−11^
MHC-II	TLR 2 (2Z7X)	−14.7	1.7 × 10^−11^
	TLR 4 (4G8A)	−16.0	2.0 × 10^−12^

PDB = Protein Data Bank.

## Data Availability

Not applicable.

## Supplementary Materials

The following supporting information can be downloaded at: https://www.mdpi.com/article/10.3390/life12050746/s1, Figure S1: Consensus enolase of the *T. cruzi* modeling results; Figure S2: Ramachandran’s graphs corresponding to enolase consensus; Figure S3: Visualization of the predicted epitopes in the enolase consensus structure; Figure S4: MHC-I protein chimeric construct modeling with AlphaFold; Figure S5. MHC-II protein chimeric construct modeling with AlphaFold; Table S1: DTUs analyzed to obtain the consensus enolase sequence [73,74,75,76,77,78,79,80,81].
