# Supplementary material for: Consensus Enolase of Trypanosoma Cruzi: Evaluation of Their Immunogenic Properties Using a Bioinformatics Approach
Source: Life (Basel). 2022 May 18;12(5):746. doi: 10.3390/life12050746 (PMC9148029; doi:10.3390/life12050746)
Supplement: Supplementary file 1 [file life-12-00746-s001.zip › life-1705592-supplementary.pdf]

Article

Consensus Enolase of *Trypanosoma Cruzi*: Evaluation of Their Immunogenic Properties Using a Bioinformatics Approach

Alejandro Diaz-Hernandez [1], Maria Cristina Gonzalez-Vazquez [2], Minerva Arce-Fonseca [3], Olivia Rodríguez-Morales [3], Maria Lilia Cedillo-Ramirez [1] and Alejandro Carabarin-Lima [1,*]

[1] Centro de Investigaciones en Ciencias Microbiológicas, Instituto de Ciencias, Benemérita Universidad Autónoma de Puebla, 14 Sury Avenida San Claudio, Ciudad Universitaria, Puebla 72570, Mexico; alejandro.diazhernandez@viep.com.mx (A.D.-H.); lilia.cedillo@correo.buap.mx (M.L.C.-R.)

[2] Herbario y Jardín Botánico Universitario, Benemérita Universidad Autónoma de Puebla, Ciudad Universitaria, Puebla 72570, Mexico; maria.gonzalezvazquez@viep.com.mx

[3] Departamento de Biología Molecular, Instituto Nacional de Cardiología "Ignacio Chávez", Juan Badiano No. 1, Col. Sección XVI, Tlalpan, México City 14080, Mexico; minerva.arce@cardiologia.org.mx (M.A.-F.); olivia.rodriguez@cardiologia.org.mx (O.R.-M.)

* Correspondence: alejandro.carabarin@correo.buap.mx; Tel.: +52-222-2295-500 (ext. 3965)

Citation: Diaz-Hernandez, A.; Gonzalez-Vazquez, M.C.; Arce-Fonseca, M.; Rodríguez-Morales, O.; Cedillo-Ramirez, M.L.; Carabarin-Lima, A. Consensus Enolase of *Trypanosoma Cruzi*: Evaluation of Their Immunogenic Properties Using a Bioinformatics Approach. *Life* **2022**, *12*, x. https://doi.org/10.3390/xxxxx

Academic Editor: Yudong Cai

Received: 14 April 2022
Accepted: 13 May 2022
Published: date

Publisher's Note: MDPI stays neutral with regard to jurisdictional claims in published maps and institutional affiliations.

Copyright: © 2022 by the authors. Licensee MDPI, Basel, Switzerland. This article is an open access article distributed under the terms and conditions of the Creative Commons Attribution (CC BY) license (https://creativecommons.org/licenses/by/4.0/).

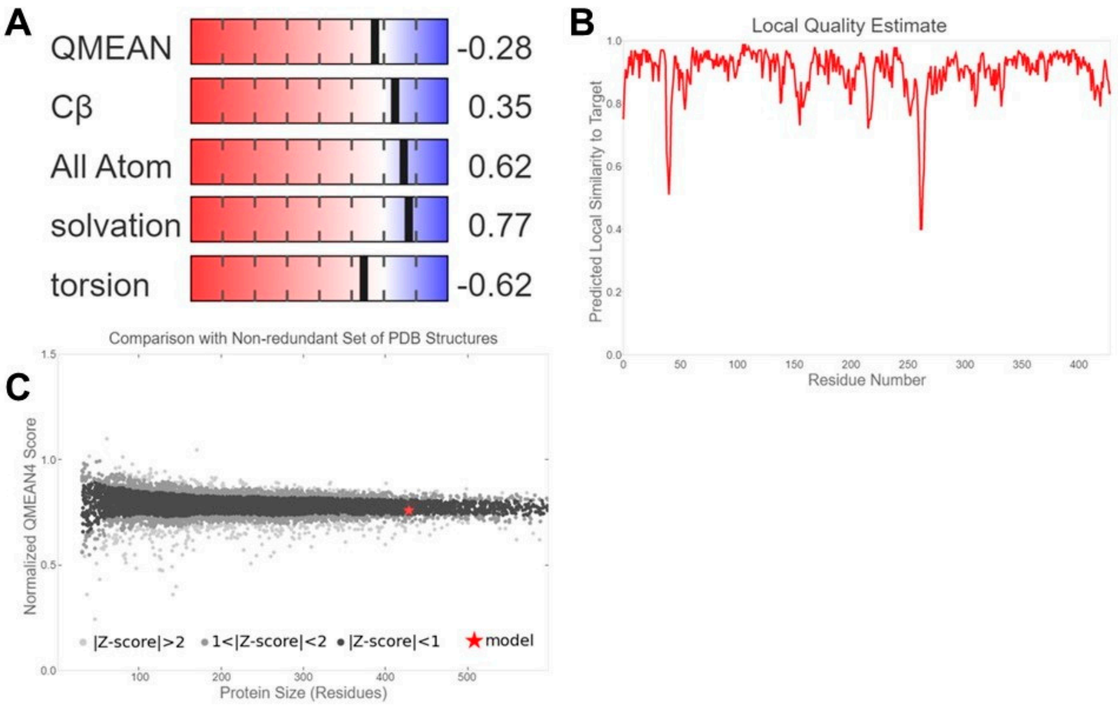

Figure S1. Consensus enolase of *T. cruzi* modelling results: A. General values obtained; **B.** Local quality plot between the model and the crystallized structure; **C.** Comparison of the QMEAN of the modeled protein with other experimentally obtained structures.

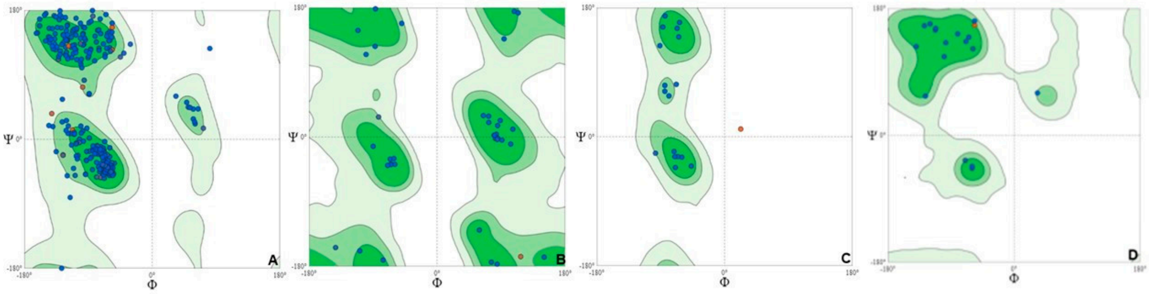

MolProbity Score	0.93	
Clash Score	0.61	
Ramachandran Favoured	96.49%	
Ramachandran Outliers	0.70%	A322 VAL, A400 ARG, A264 PRO
Rotamer Outliers	0.87%	A345 ASN, A145 VAL, A316 VAL
C-Beta Deviations	5	A52 ASP, A373 SER, A316 VAL, A264 PRO, A400 ARG

Bad Bonds	1 / 3317	A7 HIS
Bad Angles	20 / 4483	A13 ASP, (A213 PRO-A214 PRO), A264 PRO, (A220 GLU-A221 PRO), A297 ASP, (A74 ALA-A75 PRO), A31 PHE, A234 HIS, A70 ASN, (A222 LEU-A223 PRO), A253 ASN, A98 THR, A420 HIS, A7 HIS, A188 HIS, A152 ASN, A44 HIS
Cis Non-Proline	1 / 410	(A42 GLY-A43 ILE)
Twisted Prolines	1 / 18	(A263 SER-A264 PRO)

Results obtained using MolProbity version 4.4

Figure S2. Ramachandran's graphs corresponding to consensus enolase: A. General graph (without proline and glycine); **B.** Exclusive glycine graphic; **C.** Exclusive proline graphic; **D.** Graph of amino acids immediately preceding proline in the sequence

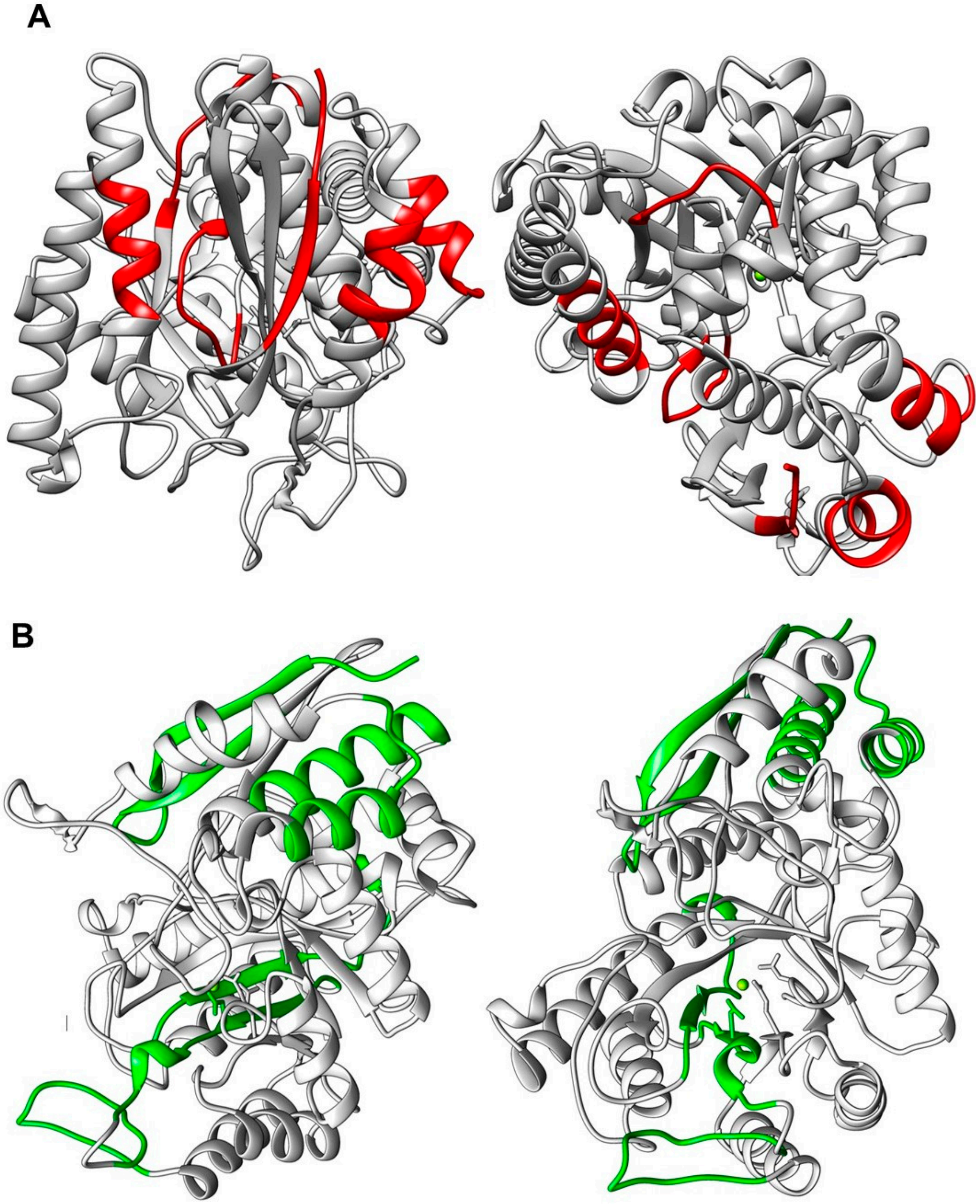
A
B

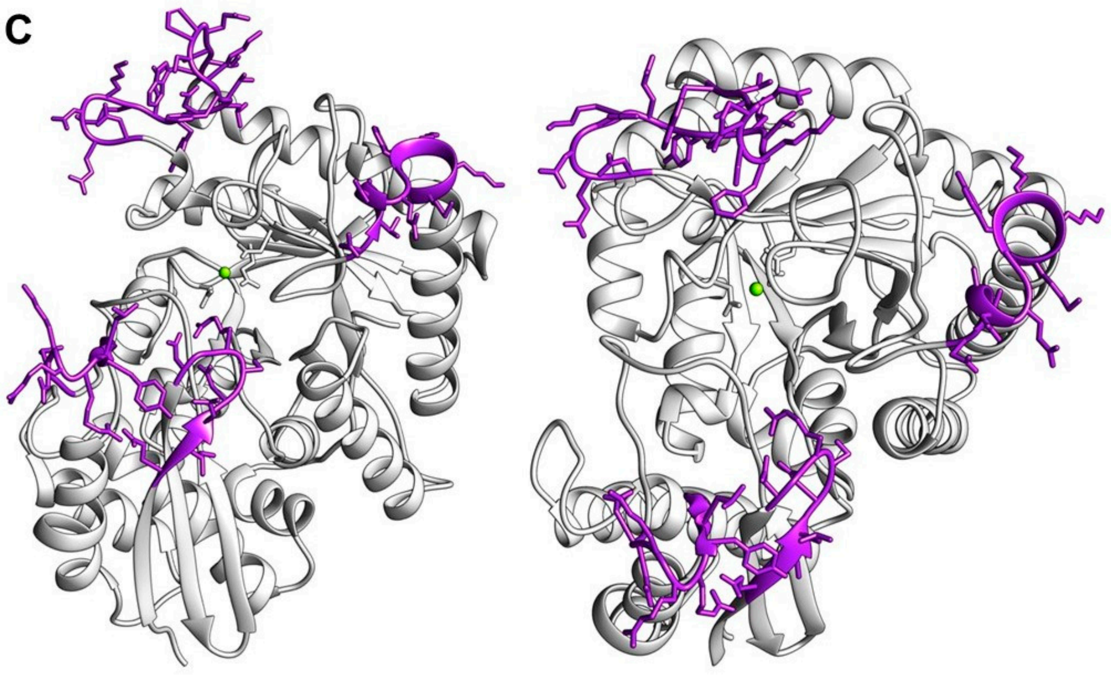

Figure S3. Visualization of predicted epitopes in the consensus enolase structure: A. Predicted MHC-I epitopes highlighted in red, showing six epitopes detailed in Table 4. **B.** Predicted MHC-II epitopes highlighted in green, showing seven epitopes as detailed in Table 4. **C.** Predicted epitopes for B cells highlighted in purple, showing five epitopes, detailed in Table 5.

Figure S4

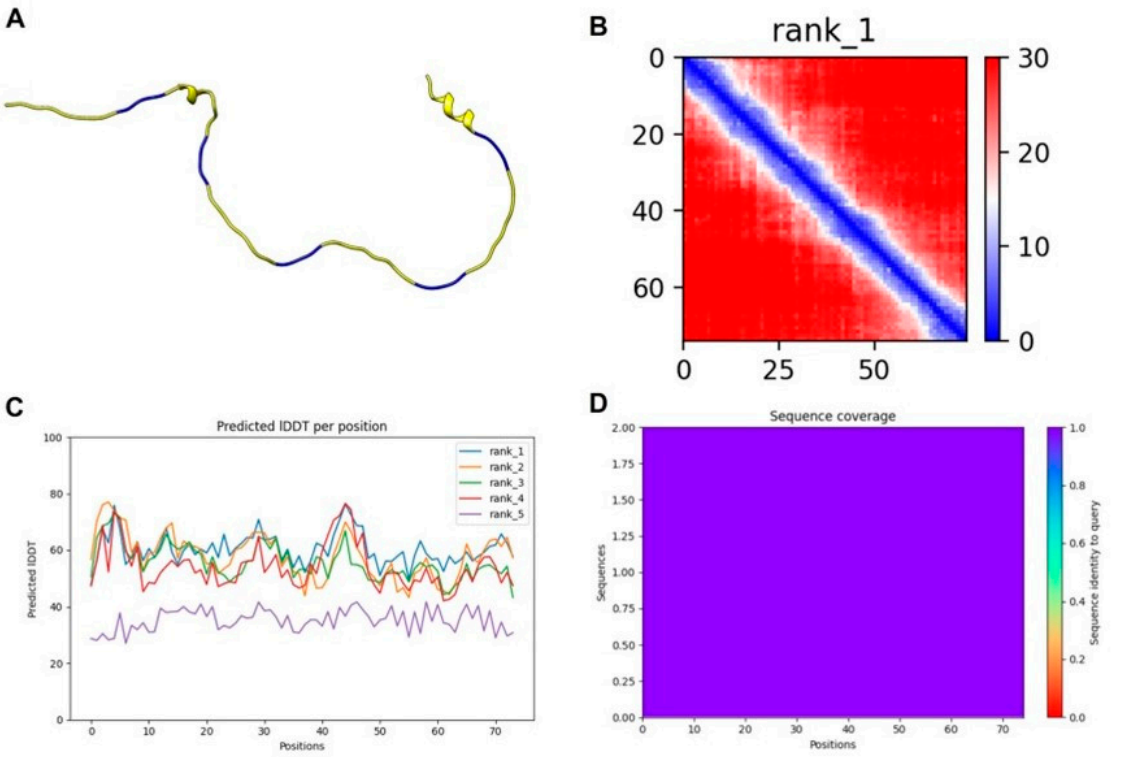

Figure S4. MHC I protein chimeric construct modelling with AlphaFold. A. Structure obtained corresponding to the MHC I type chimera, yellow for epitopes and blue for linker type proteins); **B.** Distogram with the number of sequences per position between pairs of residues (it indicates the spatial relationship, bluer is more reliable); **C.** LDDT Score of the 5 models created (values above 90 are ideal); **D.** Number of sequences per position (This graph shows us sequences found that in parts follow the same order as the Problem sequence).

Figure S5

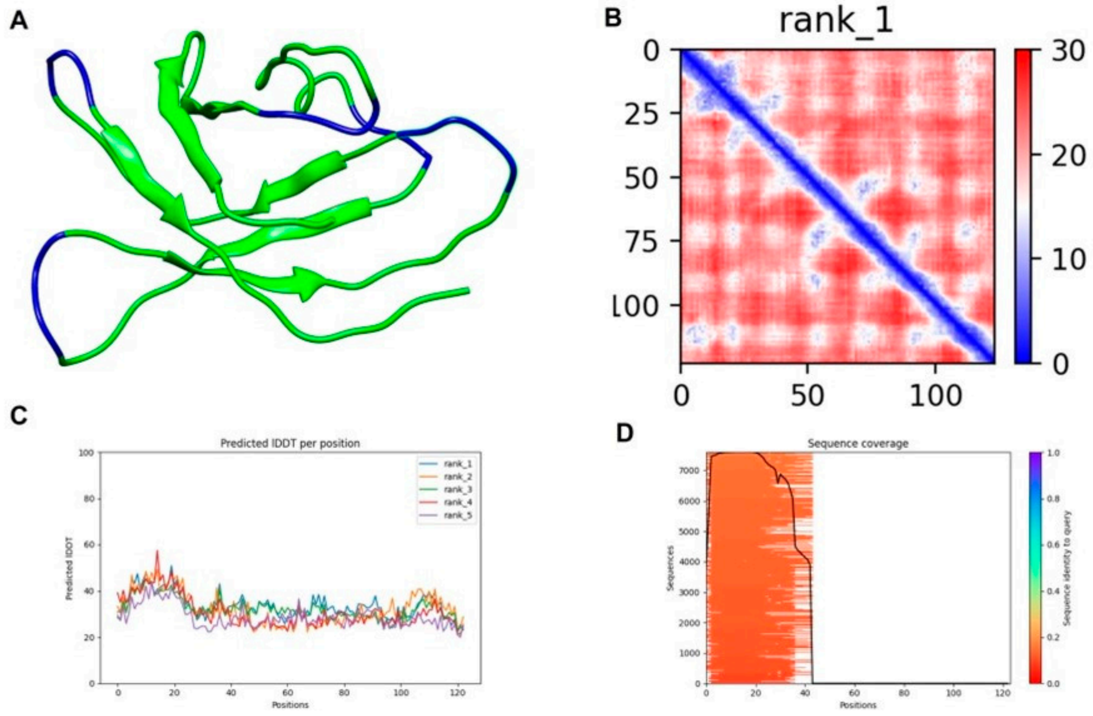

Figure S5. MHC II protein chimeric construct modelling with AlphaFold. A. Structure obtained corresponding to the MHC II type chimera, green for epitopes and blue for linker type proteins); **B.** Distogram with the number of sequences per position between pairs of residues (it indicates the spatial relationship, bluer is more reliable); **C.** LDDT Score of the 5 models created (values above 90 are ideal); **D.** Number of sequences per position (This graph shows us sequences found that in parts follow the same order as the Problem sequence).

Table S1. DTUs analyzed to obtain a consensus enolase sequence.

DTU 1	Link	Reference
Dm 28c	tritrypdb.org/tritrypdb/app/record/dataset/DS_59b1d9cd42	[1]
Jrc1 4	tritrypdb.org/tritrypdb/app/record/dataset/DS_72f799a558	N/R
Sylvio X10/1	tritrypdb.org/tritrypdb/app/record/dataset/DS_45e8b704c2	[2]
Brazil A4	tritrypdb.org/tritrypdb/app/record/dataset/DS_610b8f99b0	[3]
H8	www.ncbi.nlm.nih.gov/nuccore/KC862322.1	[4]
**DTU 2**		
Esmeraldo	tritrypdb.org/tritrypdb/app/record/dataset/DS_467358987e	N/R
Y	tritrypdb.org/tritrypdb/app/record/dataset/DS_9ddef8c95e	[5]
YC6	tritrypdb.org/tritrypdb/app/record/dataset/DS_9540582c52	[3]
**DTU 3**		
231	tritrypdb.org/tritrypdb/app/record/dataset/DS_4405580551	[6]
**DTU 5**		
Bug2148	tritrypdb.org/tritrypdb/app/record/dataset/DS_6a49629990	[7]
**DTU 6**		
TCC	tritrypdb.org/tritrypdb/app/record/dataset/DS_f35827c3d2	[1]
Marinkellei B7	tritrypdb.org/tritrypdb/app/record/dataset/DS_b0489ab98b	[2]
TulaCI2	tritrypdb.org/tritrypdb/app/record/dataset/DS_4900730c76	[8]
CL Brener non-esmeraldo -like	tritrypdb.org/tritrypdb/app/record/dataset/DS_f8bddf3a97	[9]
Cl Brener	www.ncbi.nlm.nih.gov/nuccore/XM_814607.1/	[10]

N/R= No Reference

References

1. Berna, L.; Rodriguez, M.; Chiribao, M.L.; Parodi-Talice, A.; Pita, S.; Rijo, G.; Alvarez-Valin, F.; Robello, C. Expanding an expanded genome: long-read sequencing of Trypanosoma cruzi. *Microb. Genom.* **2018**, *4*, doi:10.1099/mgen.0.000177.
2. Franzen, O.; Talavera-Lopez, C.; Ochaya, S.; Butler, C.E.; Messenger, L.A.; Lewis, M.D.; Llewellyn, M.S.; Marinkelle, C.J.; Tyler, K.M.; Miles, M.A.; et al. Comparative genomic analysis of human infective Trypanosoma cruzi lineages with the bat-restricted subspecies T. cruzi marinkellei. *BMC Genomics* **2012**, *13*, 531, doi:10.1186/1471-2164-13-531.
3. Wang, W.; Peng, D.; Baptista, R.P.; Li, Y.; Kissinger, J.C.; Tarleton, R.L. Strain-specific genome evolution in Trypanosoma cruzi, the agent of Chagas disease. *PLoS. Pathog.* **2021**, *17*, e1009254, doi:10.1371/journal.ppat.1009254.
4. Carabarin-Lima, A.; Rodriguez-Morales, O.; Gonzalez-Vazquez, M.C.; Baylon-Pacheco, L.; Reyes, P.A.; Arce-Fonseca, M.; Rosales-Encina, J.L. In silico approach for the identification of immunological properties of enolase from Trypanosoma cruzi and its possible usefulness as vaccine in Chagas disease. *Parasitol. Res.* **2014**, *113*, 1029–1039, doi:10.1007/s00436-013-3737-0.
5. Callejas-Hernandez, F.; Rastrojo, A.; Poveda, C.; Girones, N.; Fresno, M. Genomic assemblies of newly sequenced Trypanosoma cruzi strains reveal new genomic expansion and greater complexity. *Sci. Rep.* **2018**, *8*, 14631, doi:10.1038/s41598-018-32877-2.
6. Baptista, R.P.; Reis-Cunha, J.L.; DeBarry, J.D.; Chiari, E.; Kissinger, J.C.; Bartholomeu, D.C.; Macedo, A.M. Assembly of highly repetitive genomes using short reads: the genome of discrete typing unit III Trypanosoma cruzi strain 231. *Microb. Genom.* **2018**, *4*, doi:10.1099/mgen.0.000156.
7. Callejas-Hernandez, F.; Girones, N.; Fresno, M. Genome Sequence of Trypanosoma cruzi Strain Bug2148. *Genome. Announc.* **2018**, *6*, doi:10.1128/genomeA.01497–17.
8. Hamilton, P.B.; Lewis, M.D.; Cruickshank, C.; Gaunt, M.W.; Yeo, M.; Llewellyn, M.S.; Valente, S.A.; Maia da Silva, F.; Stevens, J.R.; Miles, M.A.; et al. Identification and lineage genotyping of South American trypanosomes using fluorescent fragment length barcoding. *Infect. Genet. Evol.* **2011,** *11*, 44–51, doi:10.1016/j.meegid.2010.10.012.

9. Weatherly, D.B.; Boehlke, C.; Tarleton, R.L. Chromosome level assembly of the hybrid Trypanosoma cruzi genome. *BMC. Genomics.* **2009,** *10*, 255, doi:10.1186/1471-2164-10-255.
10. El-Sayed, N.M.; Myler, P.J.; Bartholomeu, D.C.; Nilsson, D.; Aggarwal, G.; Tran, A.N.; Ghedin, E.; Worthey, E.A.; Delcher, A.L.; Blandin, G.; et al. The genome sequence of Trypanosoma cruzi, etiologic agent of Chagas disease. *Science* **2005**, *309*, 409–415, doi:10.1126/science.1112631.
